# Gastric Cancer Treatments and Survival Trends in the United States

**DOI:** 10.3390/curroncol28010017

**Published:** 2020-12-24

**Authors:** Kelly A. Stahl, Elizabeth J. Olecki, Matthew E. Dixon, June S. Peng, Madeline B. Torres, Niraj J. Gusani, Chan Shen

**Affiliations:** 1 Department of Surgery, College of Medicine, The Pennsylvania State University, Hershey, PA 17036, USA; kstahl@pennstatehealth.psu.edu (K.A.S.); eolecki@pennstatehealth.psu.edu (E.J.O.); mdixon2@pennstatehealth.psu.edu (M.E.D.); jpeng1@pennstatehealth.psu.edu (J.S.P.); mtorres2@pennstatehealth.psu.edu (M.B.T.); ngusani@pennstatehealth.psu.edu (N.J.G.); 2 Department of Public Health Sciences, College of Medicine, The Pennsylvania State University, Hershey, PA 17036, USA

**Keywords:** gastric cancer, guideline concordant care, treatment trends, National Cancer Database, multimodal therapy, cancer survival

## Abstract

Gastric cancer is the third most common cause of cancer deaths worldwide. Despite evidence-based recommendation for treatment, the current treatment patterns for all stages of gastric cancer remain largely unexplored. This study investigates trends in the treatments and survival of gastric cancer. The National Cancer Database was used to identify gastric adenocarcinoma patients from 2004–2016. Chi-square tests were used to examine subgroup differences between disease stages: Stage I, II/III and IV. Multivariate analyses identified factors associated with the receipt of guideline concordant care. The Kaplan–Meier method was used to assess three-year overall survival. The final cohort included 108,150 patients: 23,584 Stage I, 40,216 Stage II/III, and 44,350 Stage IV. Stage specific guideline concordant care was received in only 73% of patients with Stage I disease and 51% of patients with Stage II/III disease. Patients who received guideline consistent care had significantly improved survival compared to those who did not. Overall, we found only moderate improvement in guideline adherence and three-year overall survival during the 13-year study time period. This study showed underutilization of stage specific guideline concordant care for stage I and II/III disease.

## 1. Introduction

Gastric cancer is the fifth most common cancer, and the third leading cause of cancer deaths in the world after lung and colorectal cancer, accounting for over 783,000 deaths each year [1]. In the United States, gastric cancer accounts for 1.6% of all new cancer diagnosis and is estimated to be responsible for 11,140 cancer deaths in 2019 [1]. In the U.S., gastric cancer carries a poor prognosis with a reported five-year overall survival (OS) of 68.8% for localized disease, 31% for regional disease and 5.3% for distant disease [2].

The treatment of gastric cancer has drastically changed over the last few decades. Prior to 2000, negative margin gastrectomy with lymphadenectomy was considered the gold standard in treatment for stage I-III gastric cancer. However, in 2001, the Intergroup 0116 trial (INT-0116) showed the survival advantage of surgery and chemoradiotherapy in treating stage I-III gastric cancer [3,4,5,6,7]. The current literature and National Comprehensive Cancer Network (NCCN) guidelines recommend stage specific treatment. Stage I disease patients should receive endoscopic or surgical resection alone (surgery alone), surgery plus perioperative chemotherapy (surgery plus chemotherapy) or surgery and chemoradiotherapy (trimodality therapy) [8]. For Stage II/III disease the patients should receive surgery plus chemotherapy, or trimodality therapy [8,9]. For Stage IV disease, the patients should receive chemoradiation, systemic chemotherapy, or the best available supportive care [8]. The term trimodality refers to the receipt of surgery and chemoradiotherapy. Multiple prior retrospective reviews have evaluated treatment pattern trends and compliance for gastric cancer stage IB-III, and found that uptake and compliance were low [10,11]. However, none of these studies evaluated all stages of gastric cancer. With the advancements in the treatment of gastric cancer and the recommendation of stage specific guideline concordant care, little is known about how these guidelines have disseminated into practice for all stages of gastric cancer, how practice patterns across the U.S. have changed, and whether survival has improved. The aim of this study was to evaluate the trends in treatment and survival of patients with gastric cancer using the U.S. National Cancer Database (NCDB) data between 2004 and 2016. We sought to identify predictive factors for the receipt of guideline concordant care for stage I and II/III gastric cancer. We postulated, in concordance with new existing guidelines, that a larger percentage of patients would receive stage specific guideline concordant care and that patients will have improved three-year OS based on year of diagnosis. 

## 2. Experimental Section

This was a retrospective cohort study using data collected from the 2004–2016 NCDB. The NCDB is a collaboration sponsored by the American College of Surgeons and the American Cancer Society. The NCDB is a clinical oncology database that collects data from nearly 1500 Commission on Cancer (CoC) accredited facilities for individual patients using a standardized set of data elements and definitions. The NCDB data represents roughly 70% of all new cancer diagnoses in the U.S [12]. This study was deemed to be exempt from institutional review board approval by the Human Subject Protection Office at the Penn State College of Medicine.

In total, 155,555 staged cases of gastric adenocarcinoma (International Classification of Disease codes C16.0 to C16.9) were reported to the NCDB between 2004 and 2016. Tumor histologies included were adenocarcinoma, adenocarcinoma-not otherwise specified, carcinoma tumor, mucinous adenocarcinoma, intestinal-type adenocarcinoma, and signet ring cell carcinoma. Patients with other primary gastric tumor histology types or tumors at the gastroesophageal junction were excluded. Patients with missing demographic factors or treatment regimens were excluded from the study. Disease stage was determined by means of analytic stage which classifies patients based on the highest pathologic stage, and if this is unavailable it uses the highest clinical stage documented. Information on downstaging following neoadjuvant therapy is not available in the NCDB and therefore was not addressed. Given that stage II and III disease have the same treatment algorithm in NCCN guidelines, these stages were analyzed together [8]. 

A bivariate analysis was completed and compared patient demographics, facility related, tumor related and treatment related features by either stage I, stage II/III or stage IV disease based on patients American Joint Committee on Cancer (AJCC) clinical stage group per the AJCC Staging Manual Editions 6 and 7 (Table 1). Patient characteristics evaluated included sex, age, race, Charlson-Deyo Comorbidity Index (CDCI) [13], year of diagnosis, insurance status, median household income quartile from 2008–2012, and urban/rural status of home residency. Urban/rural status was divided into metropolitan, rural adjacent to metropolitan area and rural, based on the patient’s home zip code. For facility related features we included facility type, facility location by U.S. Census region, and facility case volume by quartile. Facility type was categorized as Academic/Research Cancer Programs, Comprehensive Community Hospital Cancer Programs and Community Cancer Programs. Tumor related features included tumor location, and histologic grade. Tumor location was categorized into: proximal, body, distal or unknown. Histologic Grade was categorized into: high grade, low grade or unknown. Treatment related features included types of treatment received were categorized as follows: no therapy, chemotherapy only, radiation only, chemoradiation, surgery alone, surgery plus chemotherapy, surgery plus radiation, or trimodality. The timing of treatment sequence (i.e adjuvant or neoadjuvant) was not individually analyzed. Other surgical variables evaluated in Stage I and Stage II/III patients who underwent surgical resection included surgical margins (R0, R1, R2, unknown), and adequacy of lymph node evaluation (none, unknown, <15 lymph nodes, 15–25 lymph nodes or >25 lymph nodes) (Table 2). Starting in 2010, the NCDB included a variable for the type of surgical approach. Therefore, we performed a subset analysis of patients who underwent surgical resection between 2010 and 2016 (Table 3).

We analyzed subgroup differences using chi-square test for categorical variables. For multivariate analysis, logistic regression was used to analyze the patients in the Stage I (Table 4) and Stage II/III (Table 5) cohort in order to identity the factors that were associated with the receipt of stage specific guideline concordant care. In this study we defined stage specific guideline concordant care as: surgery alone, surgery plus chemotherapy or trimodality therapy for Stage I disease and surgery plus chemotherapy, surgery plus radiation or trimodality therapy for Stage II/III disease [8]. The Kaplan–Meier method was used to assess 3-year OS for Stage I, II/III and IV disease. Five-year OS was not assessed given inadequate follow-up data for patients diagnosed in the most recent years. All statistical tests were 2-sided and alpha was set at a significance of 0.05. All analyses were performed using SAS statistical software (version 9.4).

## 3. Results

### 3.1. Patient Characteristics

During the period studied, we identified 108,150 patients with staged gastric adenocarcinoma in the NCDB. Of these 23,584 were Stage I, 40,216 were Stage II/III and 44,350 were Stage IV. Patient characteristics by stage are summarized in Table 1. Surgical variables examined for stage I and stage II/III are summarized in Table 2 and Table 3. Amongst all stages there was a predominance of male patients, White non-Hispanics, none/few comorbidities on the CDCI, insured patients, and patients who lived in a metropolitan region (Table 1). Of note regardless of stage the majority of patients (50.1–57.4%) were treated at Academic/Research Cancer Programs and a majority of these centers (55.7–64.7%) were in the 75th–100th quartile for case volume. A majority of Stage I patients (56.2%) were noted to be >70 years old while Stage II/III and Stage IV patients were aged 50–69 years old (46.7%, 48.3%). Regardless of stage the highest percentage of tumors were located in the proximal stomach. For Stage I patients, 45.5% of tumors were noted to be of low histologic grade, whereas for stage II/III and stage IV disease a majority of tumors were noted to have high histologic grade (61.1%, 57.0%).

To better determine the quality of surgery received we accessed the number of lymph nodes examined at the time of surgery as well as the surgical margins (Table 2) and the surgical approach (Table 3). Overall, 45,961 patients underwent surgical resection between 2004 and 2016. Of these 23,584 were stage I and 40,261 were stage II/III. The results showed that 46.8% of stage I patients and 44.9% of stage II/III patients had fewer than recommended number of lymph nodes examined at the time of surgery (<15 lymph nodes). Of stage I patients 23.4% had between 15–25 lymph nodes examined and 10.6% had >25 lymph nodes examined at the time of surgery. For stage II/III disease 33.9% had between 15–25 lymph nodes examined and 17.3% had >25 lymph nodes examined at the time of surgery. Upon evaluation of surgical margins, 91.7% of stage I and 81.5% of stage II/III had R0 Resections (Table 2).

Starting in 2010, the surgical approach was listed in NCDB for patients. Therefore, we evaluated the surgical approach for patients who received surgical resection between 2010 and 2016. Overall, there were 27,680 patients diagnosed with gastric cancer, 9659 patients had stage I disease and 18,021 had stage II/III disease. Regardless of stage the highest percentage of patients underwent an open procedure. However, of patients with stage I disease 34.1% underwent endoscopic or laparoscopic surgery compared to only 16% of stage II patients who underwent endoscopic or laparoscopic surgery (Table 3).

### 3.2. Evaluation of Treatment Trends over Time

**Figure 1:**
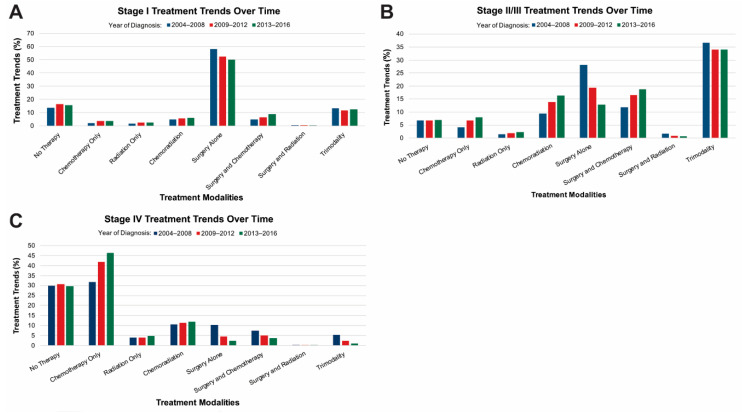
Stage I gastric cancer treatment trends by percentage, stratified by year of diagnosis; 2004–2008, 2009–2012 or 2013–2016. (**A**) For patients with stage I disease there was a decreasing trend in receiving surgery alone and an increasing trend in receiving surgery and chemotherapy. The percentage of people receiving trimodality therapy remained roughly stable over this time period. (**B**) For patients with stage II/III disease there was an increased trend toward the receipt of chemoradiation as well as surgery and chemotherapy. A decreasing trend toward receiving surgery alone. The percentage of people receiving surgery and radiation as well as trimodality therapy remained roughly stable over this 13-year time period. (**C**) Stage IV gastric cancer treatment trends by percentage, stratified by year of diagnosis; 2004–2008, 2009–2012 or 2013-2016. For patients with stage IV disease there was an increased trend toward the receipt of chemotherapy. Overall surgical treatments declined over this time period in all categories; surgery alone, surgery and chemotherapy, surgery and radiation and trimodality therapy.

Treatment trends from 2004–2016 were evaluated for each stage group. Year of diagnosis was divided into 2004–2008, 2009–2012 and 2013–2016 due to greater number of patients being diagnosed in the later years. When evaluating patients with stage I disease (Figure 1A), there was an increased trend over time toward patients receiving surgery plus chemotherapy from 5.1% (2004–2008) to 8.9% (2013–2016). Of note, during this time period, the percentage of patients who received trimodality therapy remained stable at 13.4% (2004–2008) to 12.5% (2013–2016.) The number of patients receiving surgery alone, however, decreased from 58.2% (2004–2008) to 50.0% (2013–2016).

For patients with stage II/III disease (Figure 1B), there was an increased trend towards the receipt of chemoradiation from 9.4% (2004–2008) to 16.4% (2013–2016). There was a decreased trend toward receiving surgery alone, 28.1% (2004–2008) to 12.9% (2013–2016). An increased trend towards the receipt of surgery and chemotherapy, 11.9% (2004–2008) to 18.7% (2013–2016). Finally, the percentage of patients who received surgery and radiation as well as trimodality therapy remained stable over this 13-year time period.

For patients with stage IV disease during this 13-year time period, there was a trend towards increased receipt of chemotherapy from 31.8% (2004–2008) to 46.4% (2013–2016). Overall surgical treatment declined over the years for stage IV disease in all four categories; surgery alone, surgery plus chemotherapy, surgery plus radiation, and trimodality (Figure 1C).

### 3.3. Multivariate Analyses of Predictors of the Receipt of Non-Guideline Concordant Care for Stage I and Stage II/III Gastric Cancer

Of the 23,584 patients with stage I disease, 17,242 or 73% of patients received stage I guideline concordant care, defined here as surgery alone, surgery and chemotherapy or trimodality therapy [8]. Therefore, 6221 or 27% of patients with stage I disease, received treatment that did not adhere to published guidelines (Table 4). Specifically, 15.3% of the patients received no therapy, 3.2% received chemotherapy, 2.3% received radiation alone, 5.6% received chemoradiation, while 0.5% received surgery and radiation. We found that predictors of non-guideline concordant care included African American race, age > 70, receipt of care at a facility with a lower-case volume quartile, tumor in the proximal stomach or tumor with a high or unknown histologic grade (*p* value < 0.0001 for all).

Of the 40,216 patients with stage II/III disease, 20,450 or 51%, received stage II/III guideline concordant care, defined here as surgery and chemotherapy, surgery and radiation or trimodality therapy [8]. Therefore, 19,766 or 49% of patients with stage II/III disease, received treatment that did not adhere to published guidelines (Table 5). Predictors of non-guideline concordant care for stage II/III disease included African American race, age > 70, female sex, diagnosis in 2004–2008, receipt of care in the West and Pacific, receipt of care at a facility with a lower-case volume quartile, tumor in the proximal stomach, and tumor with unknown histologic grade (*p* value < 0.0001 for all).

### 3.4. Kaplan Meier Curves for 3-Year Overall Survival for All Stages of Disease

**Figure 2:**
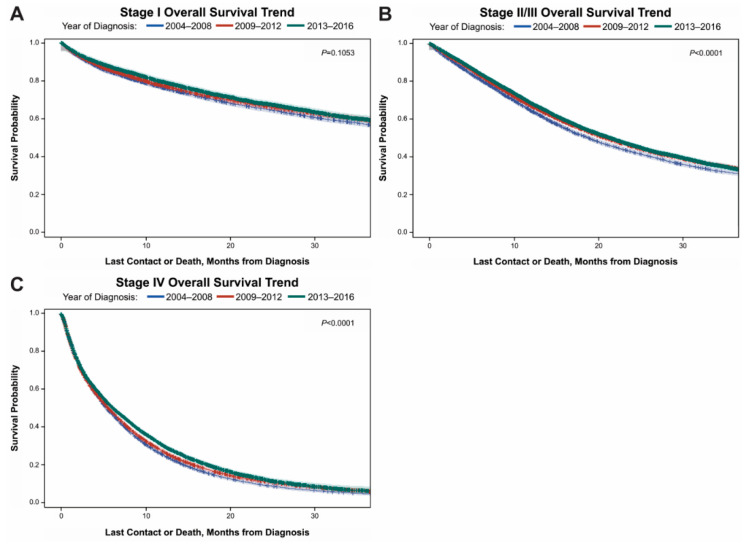
Kaplan-Meier analysis of all stages of gastric cancer 3-year overall survival stratified by year of diagnosis; 2004–2008, 2009–2012 or 2013–2016. For Stage I (**A**), Stage II/III (**B**) and Stage IV (**C**) survival is best when diagnosed in 2013–2016 and worst when diagnosed in 2004-2008 although not statistically significant.

Patients with stage I and stage II/III disease had significantly better three-year OS compared to patients with stage IV disease. Patients with stage I disease (Figure 2A) had a three-year OS of 60% in 2004–2008 that improved to 63% in 2013–2016, however this was found to not be statistically significant (*p* value 0.1053). For patients with stage II/III disease (Figure 2B), their three-year OS of 34% in 2004–2008 improved to 38% in 2013–2016 (*p* value < 0.0001). Finally, for patients with stage IV disease (Figure 2C) their three-year OS of 7% in 2004–2008 improved to 10% in 2013–2016 (*p* value < 0.0001).

### 3.5. Kaplan Meier Curves for 3-Year Overall Survival for Stage I and Stage II/III Disease Based on Receipt of Guideline Concordant Care

**Figure 3:**
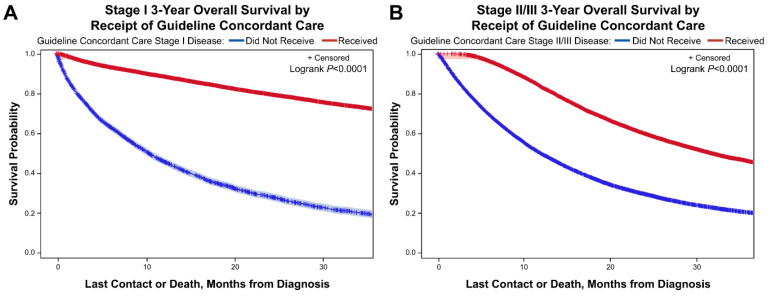
Kaplan-Meier analysis of 3-year overall survival for gastric cancer stratified by receipt or non-receipt of guideline concordant. For Stage I disease (**A**) and Stage II/III disease (**B**) survival is significantly better when patients received stage specific guideline concordant care.

Regardless of stage, patients that received guideline concordant care had significantly improved three-year OS compared to patients that did not receive guideline concordant care. Patients with Stage I disease (Figure 3A) had a three-year OS of 74% if they received guideline concordant care compared to a 20% three-year OS rate if they did not receive guideline concordant care (*p* value < 0.0001). For patients with stage II/III disease (Figure 3B) who received guideline concordant care their three-year OS was 47% compared to 20% three-year OS for those who did not receive guideline concordant care (*p* value < 0.0001).

## 4. Discussion

This study aimed to evaluate the trends of treatment and survival of patients with gastric cancer using the U.S. NCDB data from 2004–2016. In this large, multi-institutional study, we found that three-year OS has improved for stage II/III disease and stage IV disease based on year of diagnosis over this 13-year time period. We also found that three-year OS significantly improved for patients with stage I and stage II/III disease that received stage specific guideline concordant care compared to patients that did not receive stage specific guideline concordant. We found that there was an increasing trend toward the receipt of surgery and chemotherapy for stage I and stage II/III disease during this time period. (stage I: 5.1% in 2004–2008 to 8.9% in 2013–2016; stage II/III: 11.9% in 2004–2008 to 18.7% in 2013–2016). This increase was expected based on several trials that showed the benefit of adding chemotherapy to the treatment of gastric patients who received surgery [14,15,16,17,18]. The MAGIC trial demonstrated improvement in five-year OS (35% vs. 23%) in patients who received perioperative chemotherapy compared to surgery alone [17]. The use of dual modality therapy was further established with the CLASSIC trial that showed an improvement in five-year OS (78% vs. 69%) in patient that received adjuvant chemotherapy after D2 resection compared to patients who received surgery alone [16,19]. Most recently the French FNCLCC/FFCD 9703-3 study also demonstrated improved five-year OS (38% vs. 24%) in patients who received perioperative cisplatin and fluorouracil [18]. Therefore, our findings are consistent with the dissemination of recent trial results. However, our results also reflect similar findings of underutilization of guideline concordant care as those noted in Liu et. al, who found that fewer than one in three patients with stage II/III disease are receiving the neoadjuvant chemotherapy in addition to surgical resection [20]. In the context of this paper, it is important to highlight international guideline differences between the United States, Europe, and Asia. For stage I disease, the guidelines are relatively similar across the U.S., Europe, and Asia with the recommendation for endoscopic resection. However, mucosal resection is more commonly performed in the U.S. and Europe compared to Asia where both mucosal and submucosal resections are more commonly performed [8,21]. For locally advanced gastric cancer a D2 lymphadenectomy is also universally recommended by guidelines in Asian countries [22], the European Society of Medical Oncology [23,24] and the NCCN [8]. However, in the US and Europe surgeons at lower volume centers tend to perform a D1 lymphadenectomy over a D2 lymphadenectomy secondary to the increased morbidity and mortality risk ([21]. ESMO recommends if a formal D2 resection cannot be completed then at minimum 14 lymph nodes need to be removed, optimally 25 [25]. This is slightly different than the NCCN recommendations of a minimum of 15 lymph nodes [8]. Another difference in guidelines, is the timing of systemic treatment for patients with stage II/III disease. In Asian countries, neoadjuvant chemotherapy is rarely performed, and adjuvant chemotherapy is recommended over surgery alone for stage II/III patients based on the results of the ACTS-GS and CLASSIC trial [14,16,21,26]. In Europe and the US, recommendations for stage II/III disease include neoadjuvant chemotherapy followed by surgery, or adjuvant chemotherapy, or chemoradiotherapy [8,23,25]. Finally, the recommendations for palliation in stage IV disease remain universal across guidelines in the United States, Europe and Asia [8,22,25].

Our results also demonstrate that during this 13-year time period there has been no significant change in the percentage of patients receiving trimodality therapy for stage II/III disease (36.6% in 2004–2008 vs. 34.1% in 2013–2016) compared to surgery alone (28.1% in 2004–2008 vs. 12.9% in 2013–2016). However, we found that three-year OS was significantly improved with the receipt of stage II/III guideline concordant care. The improvement in OS with trimodality therapy was initially demonstrated by the INT-0116 trial which demonstrated an improvement in overall median survival in the chemoradiotherapy group compared to the surgery alone group (36 months vs. 27 months) [3]. Therefore, our findings are again in line with the literature. With the improvements in OS in patients who received trimodality therapy, the safety of these treatments was evaluated by the TOPGEAR trial which demonstrated that preoperative chemoradiation compared to chemotherapy alone prior to surgical resection can be done safely without a significant increase in treatment toxicity [27]. Variations in the quality of surgery could also contribute to the differences in observed overall survival. Our findings highlight that only 34% of stage I and 51.2% of stage II/III patients received guideline concordant lymphadenectomies. With the literature and current guidelines recommending that for stage I disease patients should receive surgery alone, surgery plus chemotherapy or trimodality therapy, our study highlights that for patients with stage I disease only 73% of patients are receiving stage specific guideline concordant care. Concurrently for stage II/III disease the literature and current guidelines recommend that patients should receive surgery plus chemotherapy, surgery plus radiation or trimodality therapy, and again our study highlights that only 51% of patients are receiving stage specific guideline concordant care.

Our findings suggest several reasons why only 73% of stage I patients and 51% of Stage II/III patients are receiving stage specific guideline concordant care. The first important factor may be patient age. We found that for both stage I and stage II/III disease a predictor of the receipt of non-guideline concordant care was patient age > 70 years old (stage I OR 2.9, CI 2.4–3.4, *p*-value < 0.001; stage II/III OR 5.0, CI 4.6–5.5, *p* value < 0.001). In our study population a large percentage of our stage I and stage II/III disease cohorts were >70 years of age (stage I: 56.2%, stage II/III: 45.9%). Another important finding is the observed racial variations in gastric cancer care. As noted in our findings, African Americans race was predictive of the receipt of non-guideline concordant care for stage I and stage II/III disease (stage I: OR 1.4, CI 1.3–1.5, *p*-value < 0.001; stage II/III: OR 1.2, CI 1.1–1.2, *p*-value < 0.001). These findings are consistent with previous studies showing racial disparities in gastric cancer treatment [28,29]. Our results also highlight that for patients with stage II/III disease diagnosed in 2004–2008, these patients were less likely to receive guideline concordant care. We believe that this highlights the relatively slow adaptation over time of new treatments regimens for gastric cancer. This slow adaptation over time can also been observed with the utilization of endoscopic and laparoscopic techniques being used for stage I disease with our results highlighting that only 34% of cases since 2010 are being performed this way. Another explanation for this could be that physicians are providing patient-centered care rather than strictly guideline concordant care, given that this may be in their patient’s best interest given advanced age, co-morbidities or other unknown confounding factors. 

When evaluating the trends in treatment for patients with stage IV disease, our findings confirmed that the development of new chemotherapy combination regimens has resulted in more patients receiving chemotherapy or radiation over this 13-year time period. With these advances there has also been an increase in the three-year OS for patients with stage IV disease dependent on year of diagnosis (7% in 2004–2008 to 10% in 2013–2016), which is in line with the Surveillance, Epidemiology, and End Results Program 2018 (SEER18) data from 2009–2015 that showed <5% five-year OS [30]. Of note the above study based on SEER18 did not comment on trends over time.

Our study should be interpreted with the following limitations. First, the NCDB only captures 70% of all CoC-approved programs within the U.S. Therefore, we cannot account for the 30% of CoC programs that are not captured, or for variations in practice at non-CoC approved programs. Secondly, patients with unstaged cancer or patients who were missing demographic, facility-related, tumor-related or treatment related variables were not included in this study, therefore this may represent a form of selection bias. However, the exclusion of patients with missing variables was limited to the smallest number of variables as to capture the largest and most accurate patient population. Thirdly certain unmeasured confounders that contributed to patients not receiving guideline concordant care, such as those who refused, those who were too sick or those who died before they could receive all forms of treatment, could have influenced the results of our data. Unfortunately, the NCDB does not have a variable that highlights the reasons why systemic therapy or radiotherapy were pursued in a patient, which would have been valuable in order to determine deviations from guideline recommended care. The NCDB also does not include variables for postoperative complications, and therefore these could not be evaluated in this analysis. Fourthly, there are intrinsic limitations related to the retrospective nature of this study involving the way data were gathered, as missing or unknown variables, as well as coded and inaccurate coding could all have influenced these data.

## 5. Conclusions

In this large, multicenter study from the NCDB, we observed that for stage I and stage II/III disease there was an increased trend toward the receipt of surgery and chemotherapy, and a stable percentage of patient receiving of trimodality therapy, and minimal improvement in three-year OS based on the year of diagnosis for both stage II/III and stage IV disease over this 13-year time period. Our findings suggest that guideline concordant care for stage I and stage II/III disease for gastric cancer has continued to be underutilized in the U.S, with disparities observed by age, race and treatment facility type. Continued awareness among all facilities, particularly cancer centers, needs to be ensured so that stage specific guideline concordant care is being provided for all patients with gastric cancer.

## Figures and Tables

**Table 1:** Demographics of the Entire Cohort, *n* = 108,150.

	Stage I (*n* = 23,584)	Stage II/III (*n* = 40,216)	Stage IV (*n* = 44,350)	*p* Value
**Sex**				<0.001
Male	15,242 (64.6%)	27,772 (69.1%)	29,067 (65.5%)	
Female	8342 (35.4%)	12,444 (30.9%)	15,283 (34.5%)	
**Age**				<0.001
<50	1149 (4.9%)	2973 (7.4%)	4315 (9.7%)	
50–69	9179 (38.9%)	18,786 (46.7%)	21,407 (48.3%)	
≥70	13,256 (56.2%)	18,457 (45.9%)	18,628 (42%)	
**Race**				<0.001
White non-Hispanic	16,593 (70.4%)	28,616 (71.2%)	30,397 (68.5%)	
White Hispanic	1735 (7.4%)	3273 (8.1%)	4363 (9.8%)	
African American	3215 (13.6%)	5591 (13.9%)	6859 (15.5%)	
Asian Pacific Islander	2041 (8.7%)	2736 (6.8%)	2731 (6.2%)	
**CDCI**				<0.001
None/few comorbidities	20,520 (87%)	36,385 (90.5%)	40,316 (90.9%)	
Multiple comorbidities	3064 (13%)	3831 (9.5%)	4034 (9.1%)	
**Year of diagnosis**				<0.001
2004–2008	8076 (34.2%)	11,165 (27.8%)	15,092 (34%)	
2009–2012	7533 (31.9%)	13549 (33.7%)	13,716 (30.9%)	
2013–2016	7975 (33.8%)	15,502 (38.5%)	15,542 (35%)	
**Insurance status**				<0.001
Not Insured	459 (1.9%)	1321 (3.3%)	2444 (5.5%)	
Insured	23,125 (98.1%)	38,895 (96.7%)	41,906 (94.5%)	
**Median income quartile 2008–2012**				<0.001
$38,000	4395 (18.6%)	7627 (19%)	8825 (19.9%)	
$38,000–47,999	5348 (22.7%)	9278 (23.1%)	10,123 (22.8%)	
$480,000–62,999	6209 (26.3%)	10,649 (26.5%)	11,800 (26.6%)	
≥$63,000	7632 (32.4%)	12,662 (31.5%)	13,602 (30.7%)	
**Urban/rural status**				<0.001
Metropolitan	20,491 (86.9%)	34,282 (85.2%)	38,201 (86.1%)	
Rural Adjacent to metropolitan area	2161 (9.2%)	4137 (10.3%)	4264 (9.6%)	
Rural	932 (4%)	1797 (4.5%)	1885 (4.3%)	
**Facility type**				<0.001
Community cancer program	1945 (8.2%)	3338 (8.3%)	4554 (10.3%)	
Comprehensive community cancer program	8112 (34.4%)	14,965 (37.2%)	17,590 (39.7%)	
Academic/research cancer program	13,527 (57.4%)	21,913 (54.5%)	22,206 (50.1%)	
**Facility location**				<0.001
Northeast and Atlantic	6085 (25.8%)	9437 (23.5%)	10,326 (23.3%)	
South Atlantic and South East	6528 (27.7%)	11,123 (27.7%)	11,931 (26.9%)	
Midwest	7137 (30.3%)	12,732 (31.7%)	14,280 (32.2%)	
West and Pacific	3834 (16.3%)	6924 (17.2%)	7813 (17.6%)	
**Facility case volume by quartile**				<0.001
0th–25th	963 (4.1%)	1673 (4.2%)	2501 (5.6%)	
26th–49th	2515 (10.7%)	4752 (11.8%)	6144 (13.9%)	
50th–74th	4855 (20.6%)	8769 (21.8%)	10,981 (24.8%)	
75th–100th	15,251 (64.7%)	25,022 (62.2%)	24,724 (55.7%)	
**Treatment received**				<0.001
No therapy	3604 (15.3%)	2745 (6.8%)	13,331 (30.1%)	
Chemotherapy only	748 (3.2%)	2625 (6.5%)	17,728 (40%)	
Radiation only	546 (2.3%)	784 (1.9%)	1924 (4.3%)	
Chemoradiation	1323 (5.6%)	5464 (13.6%)	5007 (11.3%)	
Surgery alone	12,655 (53.7%)	7752 (19.3%)	2553 (5.8%)	
Surgery and chemotherapy	1603 (6.8%)	6477 (16.1%)	2399 (5.4%)	
Surgery and radiation	121 (0.5%)	396 (1%)	84 (0.2%)	
Trimodality	2984 (12.7%)	13,973 (34.7%)	1324 (3%)	
**Location of tumor within stomach**				<0.001
Proximal	10,394 (44.1%)	20,020 (49.8%)	17,785 (40.1%)	
Body	6916 (29.3%)	10,293 (25.6%)	16,352 (36.9%)	
Distal	5344 (22.7%)	7411 (18.4%)	6665 (15%)	
Unknown	930 (3.9%)	2492 (6.2%)	3548 (8%)	
**Histologic grade**				<0.001
Low grade	10,740 (45.5%)	11,461 (28.5%)	9088 (20.5%)	
High grade	9200 (39%)	24,567 (61.1%)	25,265 (57%)	
Unknown	3644 (15.5%)	4188 (10.4%)	9997 (22.5%)	

**Table 2:** Surgical Variables Examined for Stages I and II/III, *n* = 45,961.

	Stage I (*n* = 17,363)	Stage II/III (*n* = 28,598)	*p* Value
**No. of lymph nodes examined**			<0.001
None	3292 (19%)	1049 (3.7%)	
<15	8126 (46.8%)	12,835 (44.9%)	
15–25	4062 (23.4%)	9696 (33.9%)	
>25	1836 (10.6%)	4943 (17.3%)	
Unknown	47 (0.3%)	75 (0.3%)	
**Surgical margins**			<0.001
R0 resection	15,923 (91.7%)	23,300 (81.5%)	
R1 resection	404 (2.3%)	2613 (9.1%)	
R2 resection	311 (1.8%)	1985 (6.9%)	
Not applicable/unknown	725 (4.2%)	700 (2.4%)	

**Table 3:** Surgical Approach Examined for Stages I and II/III from 2010 to 2016, *n* = 27,680.

	Stage 1 (*n* = 9659)	Stage II/III (*n* = 18,021)	*p* Value
**Surgical Approach**			<0.001
Robotic Assisted	442 (4.6%)	718 (4%)	
Robotic to Open	27 (0.3%)	59 (0.3%)	
Endoscopic or Laparoscopic	3292 (34.1%)	2880 (16%)	
Endo/Lap to Open	265 (2.7%)	614 (3.4%)	
Open	4776 (49.4%)	10,794 (59.9%)	

**Table 4:** Predictors of Non-Standard of Care Received for Stage I Gastric Cancer.

	OR	Lower CL	Upper CL	*p* Value
**Race**				
White non-Hispanic	Reference			
African American	1.384	1.259	1.521	<0.0001
Asian Pacific Islander	0.744	0.651	0.850	<0.0001
White Hispanic	1.042	0.917	1.184	0.6325
**Age**				
<50	Reference			
50–69	1.015	0.855	1.206	<0.0001
≥70	2.897	2.451	3.423	<0.0001
**Gender**				
Male	Reference			
Female	1.022	0.957	1.093	0.5133
**Year of diagnosis**				
2004–2008	0.727	0.650	.848	<0.0001
2009–2012	1.014	0.940	1.093	<0.0001
2013–2016	Reference			
**Facility location**				
Northeast & Atlantic	Reference			
Midwest	0.957	0.879	1.042	0.0622
South Atlantic and South East	1.079	0.990	1.177	0.0091
West and Pacific	0.988	0.892	1.096	0.6222
**Facility case volume by quartile**				
75th–100th	Reference			
0th–25th	3.132	2.716	3.611	<0.0001
26th–49th	2.370	2.153	2.608	<0.0001
50th–74th	1.834	1.699	1.981	0.1536
**Location of tumor within stomach**				
Distal	Reference			
Body	1.460	1.335	1.597	0.2698
Proximal	1.806	1.651	1.976	<0.0001
Unknown	1.503	1.271	1.778	0.2926
**Histologic grade**				
Low Grade	Reference			
High Grade	1.845	1.718	1.981	<0.0001
Unknown	4.528	4.154	4.935	<0.0001

**Table 5:** Predictors of Non-Guideline Concordant Care for Stage II/III Gastric Cancer.

	OR	Lower CL	Upper CL	*p* Value
**Race**				
White non-Hispanic	Reference			
African American	1.161	1.088	1.239	<0.001
Asian Pacific Islander	0.864	0.791	0.944	<0.001
White Hispanic	1.025	0.946	1.110	0.5732
**Age**				
<50	Reference			
50–69	1.498	1.373	1.634	<0.001
≥70	5.026	4.604	5.486	<0.001
**Gender**				
Male	Reference			
Female	1.175	1.121	1.230	<0.001
**Year of diagnosis**				
2004–2008	1.219	1.157	1.284	<0.001
2009–2012	1.124	1.070	1.181	0.4224
2013–2016	Reference			
**Facility location**				
Northeast & Atlantic	Reference			
Midwest	0.988	0.933	1.046	<0.001
South Atlantic and South East	1.078	1.016	1.143	0.1170
West and Pacific	1.130	1.056	1.209	<0.001
**Facility case volume by quartile**				
75th–100th	Reference			
0th–25th	1.604	1.441	1.784	<0.001
26th–49th	1.452	1.359	1.552	0.0010
50th–74th	1.331	1.263	1.402	0.8965
**Location of tumor within stomach**				
Distal	Reference			
Body	1.137	1.067	1.212	0.6917
Proximal	1.278	1.201	1.359	<0.001
Unknown	1.188	1.079	1.309	0.2800
**Histologic grade**				
Low Grade	Reference			
High Grade	0.953	0.909	1.000	<0.001
Unknown	2.169	2.007	2.343	<0.001
